# Partnership-building considerations for implementation science in learning health systems: a case study of the Implementation Science Collaborative in Alberta, Canada

**DOI:** 10.3389/frhs.2024.1327395

**Published:** 2024-02-16

**Authors:** Stephanie P. Brooks, Cody Alba, Denise Thomson, Sara N. Davison, Kate Storey

**Affiliations:** ^1^ Learning Health System Team, Alberta SPOR SUPPORT Unit, Department of Medicine, University of Alberta, Edmonton, AB, Canada; ^2^ School of Public Health, University of Alberta, Edmonton, AB, Canada; ^3^ Department of Medicine, University of Alberta, Edmonton, AB, Canada

**Keywords:** implementation science, relationships, partnerships, research capacity, learning health systems, embedded research

## Abstract

**Introduction:**

Implementation of health innovations is inherently collaborative, requiring trans-sectoral partnerships between implementation researchers, innovation teams, and implementation practitioners. Implementation science has been shown to improve implementation successes; however, challenges that hinder partnerships to advance implementation science continue to persist. Using a whole-system approach to assess and respond to implementation science partnership barriers may shed light on effective responses.

**Methods:**

We conducted a case study of Alberta's learning health system, using semi-structured group and individual interviews to create a nuanced understanding of the considerations required for implementation research collaborations. We interviewed 53 participants representing 21 offices in the health system, academia, professional associations, and government who regularly plan, evaluate, and/or study health system implementation initiatives in Alberta. Using the Partnership Model for Research Capacity Building, we identified current facilitators and challenges for partnerships for conducting and using implementation science, at different levels of Alberta's health-research ecosystem.

**Results:**

Alberta's healthcare system is well set up to readily embed intervention effectiveness and efficacy research. Infrastructure was also in place to strengthen implementation practice. However, weaknesses around exchanging knowledge and skills, providing feedback and mentoring, and accommodating diversity affected the ability of both individuals and teams to build implementation science capacity. Without this capacity, teams could not participate in embedded implementation research collaborations. We report the response of the Alberta Strategy for Patient-Oriented Research SUPPORT Unit to these barriers to provide practical guidance on various program options to strengthen individual- and organization-level implementation science capacity.

**Discussion:**

This study applied a whole-system approach to assess factors across Alberta's health-research ecosystem, which affect partnerships to advance implementation science. Our findings illustrated that partnership considerations go beyond interpersonal factors and include system-wide considerations. With the results, health organization leaders have (1) a method for assessing organizational capability to readily embed implementation research and (2) a catalog of potential responses to create conditions to readily engage with implementation science in their day-to-day implementation processes.

## 1 Introduction

Increasingly, incorporating implementation science (IS) into change initiatives is recognized as a cornerstone activity of learning health systems and other health organizations committed to continuous improvement and evidence-based care (1–4). Furthermore, the importance of IS capacity has been suggested as a core competency for embedded health systems researchers (1, 5, 6) and implementation practitioners (7). IS is defined as “the scientific study of methods to promote the systematic uptake of research findings and other evidence-based practices into routine practice, and hence, to improve the quality and effectiveness of health services” (8, p. 1). As such, people working in health promotion, prevention, and healthcare embrace IS because it enables innovation teams with evidence-based strategies to best apply and sustain change in the real world (9, 10).

In the context of IS, implementation researchers are defined as people who study implementation methods and generate knowledge to promote the uptake of evidence-based policies and practices (11). However, implementation research is not simply an activity to be undertaken by individuals. To ensure the relevance and applicability of implementation research, the science should be co-produced through close collaboration between implementation researchers and implementation support practitioners (12–14) [i.e., those who use the findings from implementation research to strengthen the implementation, spread, and scale of change efforts (11)]. Indeed, academic–practice partnership is considered a key component of implementation as it contributes to closing the research-to-practice gap (13, 15, 16). Such partnerships provide opportunities to create relevant and applicable knowledge about implementation (13, 15, 16). These partnerships can be between implementers, implementation support practitioners, researchers, healthcare staff, policymakers, patients, and any other party interested in or impacted by implementing innovations. Nevertheless, current studies highlight a persistent gap between implementation research and practice, emphasizing that many implementation research partnerships lack the degree of collaboration required to create actionable implementation recommendations that can be scaled, spread, or sustained (11, 12, 16).

There is growing interest in the IS community to resolve misalignments between implementation researchers and support practitioners that limit the uptake of IS. Training models have been developed to build a cadre of highly trained implementation researchers (17, 18). Similarly, teams continue to develop implementation support practitioner competencies to facilitate the uptake of evidence-based change using IS models and frameworks (19, 20). Team and organization models have been developed to support implementation research collaboration across the academic–health research ecosystem (21–24). Less attention has been paid to the systems within which individuals and teams work, and what system enablers are required for organizational leaders and staff to readily embed implementation research. Given the importance of academic–practice partnerships in IS, understanding partnership enablers and challenges at the micro-, meso-, and macro-level of the local systems is critical to resolving the implementation research-to-practice gap.

Embedded health researchers are increasingly utilized to facilitate academic–practice partnerships. These researchers are housed as members of health service teams where they collaborate to conduct research in real-world settings (25). Through this collaboration, embedded researchers also help build healthcare professional capacity to utilize evidence as it emerges (26). Embedded health researcher models vary but often these researchers act as conduits between healthcare delivery and academic research teams or may have academic cross-appointments themselves (25, 27). Research examining embedded health researcher models in healthcare provides critical insights regarding organizational capacity to enable research collaborations (27–31). These studies highlight the characteristics of embedded research partnerships (27), including individual skills required to build research partnerships and use evidence created in embedded research (29). These areas of the literature highlight that factors throughout the local academic–health research ecosystem affect embedded research relationships [e.g., individual skills (28, 29, 32), team dynamics (27, 30), organizational research culture (31, 33, 34), organizational research infrastructure (30, 31, 33, 34), and whole system engagement (30, 31)]. These findings focus on the effect of existing enablers and challenges, rather than on preparing systems to readily embed research. Furthermore, the limited studies that examine such preparation are sector-specific (28, 30, 34, 35), leaving an ongoing gap around how to increase system readiness to embed implementation-specific research partnerships. Consequently, guidance is limited on how to enable collaboration between implementation researchers and implementation support practitioners.

For this this study, we used a whole-system approach following the definition of Komashie et al.: “…a way of addressing health delivery challenges that recognizes the multiplicity of elements interacting to impact an outcome of interest and implement[ation of] processes or tolls in a holistic way” (36, p. 2). Through this approach, our study provides insights into the factors at various system levels that impact academic–practice partnerships to advance IS. To help build an understanding of how to respond to such factors, we also describe how one organization in Alberta, Canada, chose to overcome the barriers identified in this study and strengthen the provincial health system's ability to readily embed implementation research. Our research does not aim to evaluate the initiative described. Rather, we use a single case study approach to illustrate the process of assessing and strengthening whole-system readiness to facilitate embedded implementation research partnerships and increase IS capacity.

## 2 Methods and materials

### 2.1 Study design

We used a single case study design because of its suitability for exploring complex system factors affecting research collaborations (37–40). This qualitative case study focused on partnership considerations for various potential partners involved in advancing IS in Alberta, Canada's health-research ecosystem.

### 2.2 Case characteristics

Canada is split into 10 provinces and 3 territories with some governance being the responsibility of the federal government and some of the provincial/territorial governments. Healthcare is funded by the federal government, but provinces and territories are responsible for designing, managing, and delivering health services (41). Alberta's healthcare delivery and some public health functions are managed by a single province-wide health authority, Alberta Health Services (41, 42). Alberta Health Services delivers care through provincial-level programming as well as through five regions, called zones, that provide health programs in locally relevant ways, meeting the needs of urban, rural, and remote settings (41, 42).

The provincial government and Alberta Health Services have heavily invested in health research infrastructure through Alberta Health Services’ incoming electronic health record system (43) and Strategic Clinical Networks™ (SCNs). The electronic health record is a key feature of Alberta's learning health system, as it enables real-time capture of health experiences and outcomes to support learning and improvement (1). The SCNs are large research and innovation teams embedded into the health system to facilitate the uptake of evidence-based care from piloting programs through spread, scale, and sustainment (44). The SCNs partner with provincial and zone-level program offices, operations teams, patients, academic researchers, and other interested parties to conduct research and implement change (45). SCN-University Liaisons use their cross-appointments at SCNs and different Albertan post-secondary institutions to assist with this facilitation (46). The SCN structure provides a key learning health system link between clinicians and researchers to identify and answer key questions with rigor to inform policy, practice, and funding decisions in the health system (47). The SCN staff are highly trained in quality improvement, and many team members have advanced research degrees. As such, the SCNs are understood as the engine driving Alberta's learning health system (47); however, the teams have varying experience with IS specifically.

This environment has a strong embedded research culture and supportive infrastructure in place to conduct intervention efficacy and effectiveness research. However, interest and capacity in implementation research in Alberta are fragmented (22). Besides the SCNs, Alberta also has numerous other health research networks and intermediary organizations [i.e., organizations responsible for knowledge transfer and mobilization (48)] that further support implementation practice and research in the province. One of these intermediaries is the Alberta Strategy for Patient-Oriented Research SUPPORT Unit (AbSPORU). AbSPORU is part of a national strategy funded by Canada's main health research funder, the Canadian Institutes for Health Research. AbSPORU's mandate is to build partnerships and provide research, knowledge dissemination, and implementation services that support moving evidence into practice, specifically to strengthen Alberta's learning health system (22). AbSPORU's mandate is set broadly to respond to health system needs as they emerge. Therefore, the latitude AbSPORU has to support implementation through partnerships and services, however that may look for the health system in a given moment, makes the organization an ideal host for various collaborative initiatives to advance IS (49). At the time of writing, AbSPORU’s implementation support services included implementation science training and consultations for implementation planning, evaluation, and research. AbSPORU has also hosted conferences, implementation-specific events, and collaborative discussion forums to bring together various parties interested in and impacted by implementation, build implementation partnerships, and strengthen provincial implementation initiatives. Consequently, AbSPORU facilitates implementation research and practice partnerships with the long-term aim of increasing embedded implementation research.

AbSPORU's implementation support services pre-existed this research. These supports stemmed from a needs assessment completed in 2016, reported by Thomson et al. (49). This assessment found that inaccessible IS evidence, exacerbated by deficient knowledge sharing opportunities for change agents, limited IS capacity in the province (49). In response, AbSPORU built numerous initiatives around four core needs: (1) consultation, (2) community of practice, (3) capacity-building, and (4) contributing to knowledge translation and implementation science. The current study was conducted to assess provincial changes in Alberta's health research context and inform ongoing suitability of AbSPORU programming.

### 2.3 Participants

Between August and December 2022, we conducted 21 semi-structured interviews with 53 participants representing 21 offices in academia or the health system who regularly plan, evaluate, and/or study health system implementation initiatives in Alberta (Table 1). We recruited interview participants based on responses from an online survey administered before 2 years. The original survey was distributed to collect data for a social network analysis of Alberta's implementation community (manuscript development underway). The survey was sent to people involved in planning, evaluating, and studying implementation in Alberta. The goal of this survey and social network analysis was to learn who in the province was involved in implementation support and/or research and how these actors collaborated or not. Respondents who identified that they had engaged in implementation academic–practice partnerships in Alberta were invited to participate in these follow-up interviews reported in this article.

**Table 1:** Categories of participants involved in the study.

Type of organization	Number of interviews (*n*)	Number of interview participants (*n*)
Academics	6	7
Strategic clinical networks	6	35
Provincial offices	3	3
Intermediaries	2	4
Zone offices	1	1
Government	1	1
Primary care	1	1
Research networks	1	1
Total	21	53

We held individual (*n* = 14) and group (*n* = 7) interviews. Group interviews ranged from 2 to 10 people per interview. The 21 offices interviewed represented a spectrum of experiences with IS, with the majority (*n* = 10) facilitating implementation, some (*n* = 6) participating in IS research activities, and others (*n* = 6) actively conducting IS research.

The nature of implementation work varies across the actors in Alberta's implementation community. Some people work in collaborative teams and others act as sole implementation representatives of offices, academic departments, or organizations. Because of this range, we offered to hold group or individual interviews to the participants’ preference. People who worked more independently (e.g., academic researchers) most often opted for individual interviews and those who worked on highly collaborative teams (e.g., SCNs) chose group interviews, citing the interviews as opportunities for team members to share and learn from one another. As such, the number of SCN participants appears to be over-represented; however, SCN teams were considered as one interview each, similar to how a single academic or policymaker would represent a lab or a government office, for example.

### 2.4 Data collection

Semi-structured interviews were conducted either in person or virtually over Zoom and ranged from 30 to 85 min in duration. All interviews were conducted by the lead author with a co-researcher (CA) also attending all interviews to ensure coverage of the interview guide. The same interview guide was used for all interviews. Our interview guide addressed (1) organizations’ implementation work history and capacity, (2) criteria and processes for establishing collaborations, (3) facilitators and barriers to collaborations, and (4) recommendations to strengthen future collaborations to advance IS. This guide was developed to contextualize the results of the social network analysis and inform what additional implementation infrastructure would increase engagement in IS partnerships by various potential partners in Alberta. We asked participants to reflect on past experiences collaborating for implementation practice and research to answer the interview questions. Our goal was to identify how to address challenges and strengthen IS capacity in Alberta.

Interviews were audio-recorded, transcribed verbatim, and reviewed for accuracy. We also conducted two member-checking activities to give participants opportunities to clarify their contributions, include any comments, or ask additional questions. Participants were given a summary of their interview responses before analysis to ensure we accurately captured and understood their answers to the interview questions. Feedback from the first interview summary was clerical in nature (e.g., clarifying the organization structure). We integrated all feedback into the final analysis. Participants were also given a preliminary analysis after all interviews were completed. We asked whether the analysis resonated with the participants and if they had any additional insights to add. Members from four of the interviews responded and confirmed that the analysis resonated. No one had additional feedback on the analysis.

### 2.5 Data analysis

NVivo 11 qualitative analytic software (50) was used to organize and manage transcripts of the audio-recorded interviews. Directed qualitative content analysis was used to code the interview data both inductively and deductively (51). Deductive coding was guided by the Partnership Model for Research Capacity Building (35) (referred to as the Partnership Model in the remainder of this article) to examine whether the overall system is set up to readily embed implementation research into real-world implementation initiatives. Inductive coding facilitated thematic coding for a more nuanced understanding of partnership considerations.

Each interview transcript was coded by two researchers (SB and CA) who also reviewed each transcript together to check individual biases and bring richer analytic power by analyzing the transcripts through two perspectives (52). Analytic rigor was further enhanced through regular meetings after each interview to discuss emerging findings. After each interview, the dataset was considered, and saturation was suspected nearing the end of our scheduled 21 interviews. We conducted the final two to three interviews already scheduled and confirmed saturation as no new information emerged (53). This approach to coding helped identify barriers and facilitators that could be strengthened to increase partnerships to advance IS in Alberta's health system. Moreover, individual and team-level capacity to engage in IS was assessed based on participants’ self-described historical roles in embedded implementation research.

All study participants provided informed consent to be included in this research. The research design was approved by the University of Alberta Research Information Services, Research Ethics Board—Health Panel (ID: Pro00084611).

### 2.6 Theoretical framework

**Figure 1:**
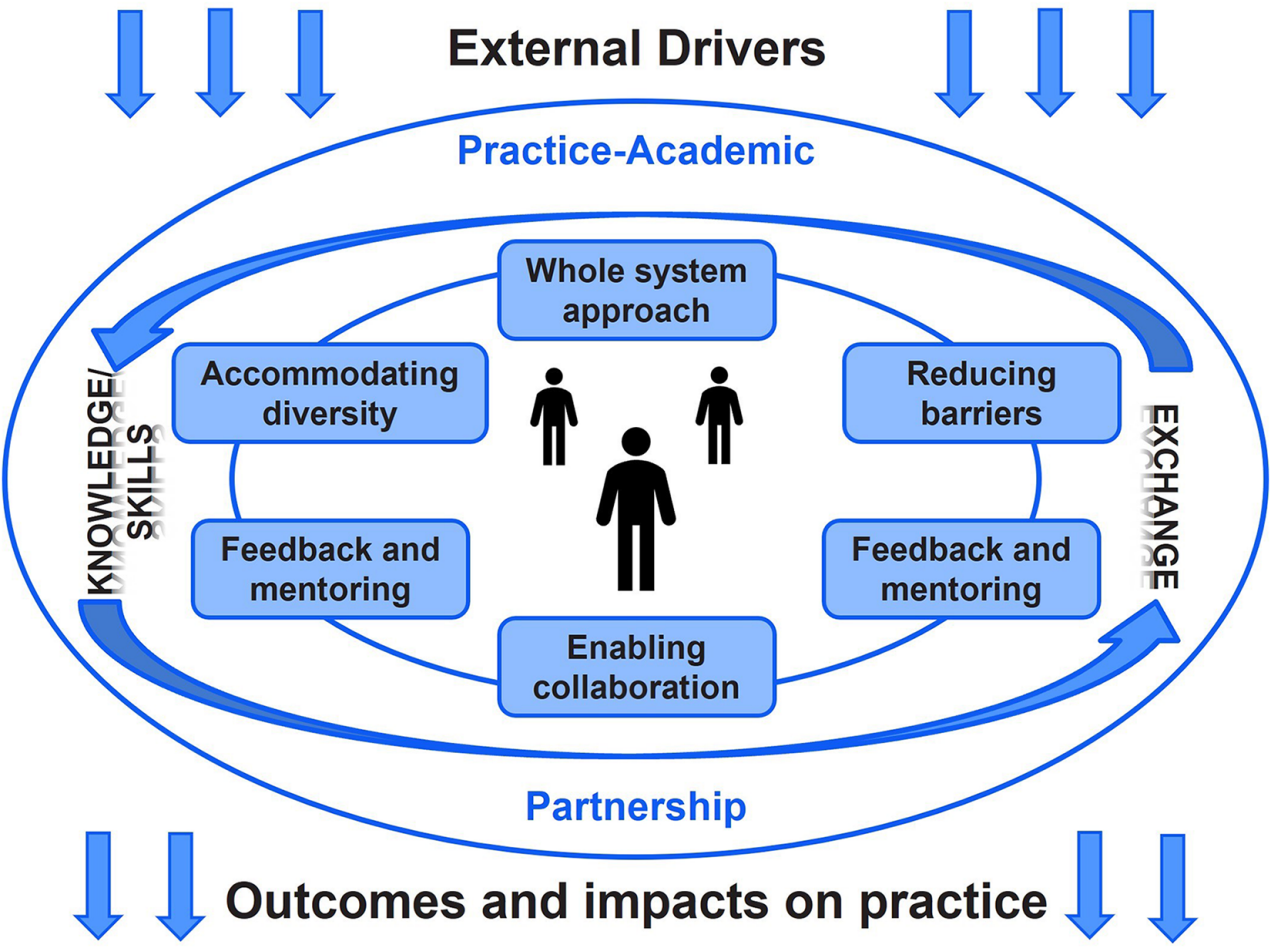
Partnership Model for Research Capacity Building (35). Permission to reprint this model was granted by BioMed Central Ltd.

The Partnership Model (Figure 1) is a theory-based model, developed to build health organization research capacity in one health professional group, speech and language therapy (35). The authors of the model emphasize that, “The need for researchers to be aware of how findings will be used and interpreted by healthcare professionals, and for the research to reflect issues relevant to those at the interface of patient care, are both paramount to successful implementation of research outcomes”, (35, p. 289). They posit that collaborative research between healthcare professionals and researchers creates this synergy by ensuring researcher relevance and healthcare professional ability to critically engage with evidence. To achieve this engagement, both individual research capacity and a research-enabling context are required. Together, these two required elements dictate the research readiness of a given health research ecosystem (35).

The model's developers recognize research relevance as vital for successful implementation. However, their work refers to intervention and health outcome research, stopping short of using the model to assess implementation-specific research readiness. The distinction between intervention and implementation research is important as knowledge from both fields inform implementation practice (54). Therefore, engagement in IS partnerships is critical for ensuring the successful uptake of health innovations (54). Despite the narrow scope of the original model, Whitworth et al. claim that the Partnership Model is transferable beyond the domain of speech and language therapy (35). This transferability was an attractive feature for our research team given the model's whole-system view of research readiness and its emphasis on practice–academic partnership. Furthermore, this framework focuses on the contextual factors that underpin an organization's research capacity–building capabilities, a key interest of AbSPORU given its capacity-building mandate. Thus, we used this framework to assess Alberta's capacity to conduct implementation research and use IS in practice. In turn, this study also enabled our team to gauge the usefulness of the Partnership Model to assess IS capacity and context.

The Partnership Model outlines essential components of effective embedded research environments. The model places particular importance on practice–academic partnership, which enables reciprocal knowledge and skills exchange across partner organizations. Knowledge and skills exchange is considered a key feature of research-ready organizations because it indicates the ability to align the values of different organizations into integrated collaborations. Research-ready systems follow six principles in their research work, represented in boxes in Figure 1, which underpin effective partnerships to increase the research capacity in healthcare settings (55). First, research-ready systems support a whole-system approach that enhances the potential for professionals at different stages of their careers identify embedded research opportunities and develop organizational research pathways. Second, accommodating the diversity of individual research interests, learning styles, and backgrounds is required to align inter-organization priorities and work processes. Third, facilitating networking opportunities between different parties involved in potential research helps link teams with similar interests who would not otherwise connect through regular day-to-day work. Fourth, enabling collaborations across system levels, sectors, and professions is especially important for organizations supporting intra- and inter-disciplinary collaborations, such as implementation research (21–24). Fifth, providing feedback and academic mentoring increases skills for planning, funding, and conducting research. Sixth, research-ready organizations make ongoing efforts to identify and overcome barriers to embedded research [e.g., build funding opportunities for priority research areas (35, 47)]. Finally, the model couches all components in external drivers and the goal of impacting practice and patient outcomes, represented by the arrows entering and exiting the rest of the model. External drivers differ by the research partner, dictating the shared focus of the collaboration, motivations to collaborate, and conditions within which each partner can contribute to a given academic–practice partnership. Proposed outcomes and impacts are related to the external factors as they create motivation, act as the basis for securing academic–practice partnership resources, and help establish research mandates.

## 3 Results

We used the Partnership Model of Whitworth et al. (35) as our analytical framework to code the data because it helped provide insights into enablers and challenges to readily embedding implementation research in Alberta's health system. We found that Alberta had many enablers in place to facilitate embedded intervention efficacy and effectiveness research, but that numerous challenges remained for embedding implementation research specifically. Below, we describe the participating teams’ and individuals’ experiences with collaborating to conduct implementation research and use IS in implementation practice. The remaining results are categorized by the components of the Partnership Model, followed by a summary of AbSPORU's various efforts to increase IS capacity across the province. Each section includes exemplar quotes. For improved readability, and where meaning remained unaffected, we removed non-lexical terms (e.g., um) and grammatical errors.

### 3.1 Participants’ existing experiences with IS collaborations

**Figure 2:**
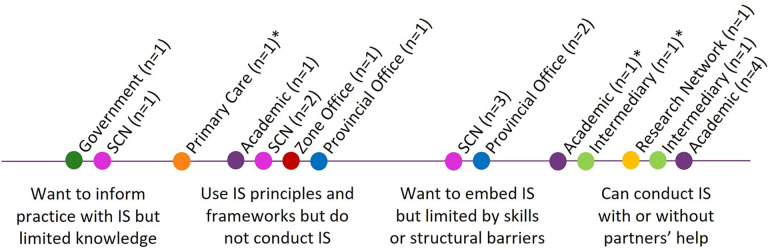
Participant exposure and comfort with IS. The professional categories are color-coded on the spectrum. *Actively working to move into the next category to the right.

The participants included implementation researchers, implementation support practitioners, intermediaries, and policymakers. All of the participants stated that they value IS, and most teams desired to engage in implementation research collaborations. However, some practice teams were not engaged in implementation research partnerships at the time of the interviews. The scope of each individual's or team's role in facilitating implementation or conducting implementation research varied. The participants also had varying experiences with IS (Figure 2).

Mapping the different teams to the spectrum in Figure 2 uncovered (1) individual and team IS capacity and (2) the strength of organizational mandates or expectations for teams to engage in IS. The spectrum highlighted participating teams’ comfort and interest in being involved in IS. At the individual and team levels, this provided important information about what types of supports could be offered to increase IS capacity (e.g., formal training, informal guidance, and mentorship programs). Mapping teams onto this spectrum also provided insights into the organization-level mandates of different teams. For example, different SCNs, indicated by pink dots, fell across the spectrum, demonstrating a weak mandate from the organization for teams to engage in IS.

### 3.2 Practice–academic partnership

Interview participants described the health-research ecosystem as one that values transdisciplinary research partnerships. However, participants recognized the “messiness” of practice–academic partnerships and the effort it takes to align the different priorities and needs of the different partners:

I do think a lot of it is culture. I think we have to try and bridge what they call two solitudes. I think we have a research thing going and we have a health system thing going, and I think we need to get those closer together. I used to talk about research practice partnerships but again, the challenge there is: how do potential academic collaborators get rewarded for working with those of us in the health system? Because it does get messy, and it does get a little bit difficult, and it can take people outside their comfort zone. But if we're thinking about young people who are coming into the system who need to establish themselves, we need to be able to uphold our end of the bargain as well (Provincial Office 2).

Building trust and strengthening relationships was paramount for teams across sectors to engage in partnerships to advance IS. Participants emphasized that building trust in relationships takes time and requires an ongoing commitment to delivering on partnership promises. Participants who worked to build relationships with health system teams said

To establish and build relationships with these teams and these individuals and to slowly build trust over time, people need to know that they can come to you, you will be helpful, and you will kind of adapt what your responses are to what their actual needs are. And that's something that takes a lot of time and is something that happens over the course of longer projects or multiple projects. That's really the biggest thing, how we as a team are able to support both the implementation support aspects and then the science aspects of the work going on (Intermediary 2).

I don't feel like you just bring someone on to bring someone on, unless can you get along … I'm very much an optimist, and I like to see the positive side in any and all people. But I've also been here for a while as an academic, and I have had negative experiences with collaborators … I tend to try to work on those relationships first … I want to get to know that person and understand them … I have a lot of really amazing partners who are open to my crazy ideas … because I've built up that trust and rapport that IS is necessary and that this should be something that we're invested in, not just doing kind of these one-offs (Academic 6).

[Collaboration] is about lots of things, but the main thing is relationships. It's that trust. What facilitates trust is always delivering, because I've heard so many times from community that, “We wrote a letter of support for their grant and then we never heard from them again … We're not sure what happened, and so we just don't want to work with them anymore, those university types because they don't deliver”. And so, one of the things that I've done is that I have always delivered. It's hard and it takes time, and I'm always late because I'm saying yes to too many things, but I always deliver (Academic 5).

### 3.3 Knowledge and skills exchange

Knowledge and skills exchange is required to build enough individual capacity to identify opportunities and partner for embedded implementation research. Sometimes people with IS experience were hired to build research capacity within their teams. As one participant shared,

So locally, it has been trying to build capacity with the members, with the primary investigators and their teams within our large team. Also, an example of building capacities is being on multiple grants with our PIs if they are related to implementation. So those have been strategies to express or demonstrate the difference between implementation research or science vs. the application of implementation and support (Academic 4).

Some participants felt that this approach was potentially helpful for capacity-building and networking, as one SCN member said

… building awareness or introducing SCN scientists to academics who are doing IS because, then perhaps there would be more integration between the academics in IS and the SCNs (SCN 4).

However, IS knowledge was most often brought to implementation initiatives in the health system through one-way consultation, rather than through reciprocal knowledge exchange. This consultation model limited capacity-building in Alberta and inadequately supported change initiatives. Participants noted that for teams requiring external IS support, consultants need to be “sufficiently embedded” to understand the context and make useful contributions to the larger change initiative. As SCN team members who had previously worked with IS consultants stated,

When the IS works, it's sufficiently embedded in the day-to-day operations of a project. Whether that's the consultants stay with the project or whether another project lead feels that you know, that's in effect, what we're doing every day is IS and it works well. And that was our experience. If you ask the leaders of that project, they would say that every part of that project is IS. It's not something that we add on or we get kind of input on periodically from an implementation scientist. It is the purpose of the project (SCN 36).

My limited experience previously, not just with Alberta Health Services but with other places as well, is that when implementation scientists fly in and fly out, to consult on a project, it doesn't work. It has to be like a journey that we go through together. Otherwise, other consultants kind of fly in, fly out and they make comments and suggestions without really fully understanding the context and the nuances of each process, then it becomes sometimes a bit confusing, and people just look at that, like, how is that helpful to us? And so, in the end, they just don't use it (SCN 28).

Participants also described the lack of cross-sectoral pathways to communicate implementation research findings or practice lessons learned to other teams in the health-research ecosystem. As one participant said,

We need some sort of platform where somebody could go, they can search for information, where they can learn and just be able to bounce ideas off of other people that have done something similar in the past. I think that's a gap we need to fill (Provincial Office 3).

A theme that ran through all of the interviews was that the terminology surrounding IS was confusing and in turn limited collaboration. Implementation facilitation, implementation support, quality improvement, dissemination, IS, and conducting IS were often conflated with one another. Participants who had successfully partnered for IS attributed a portion of their success to discussing IS in ways that resonated with partners. As one academic put it,

I've always believed that right from the start of this work, if we can't translate what we're doing as implementation or knowledge translation people, what are we doing? Even our terminology and the confusion around it. So, to me, it's key to be that facilitator and being able to translate to your audience that you're going to be talking to (Academic 4).

Participants from the health system also shared perspectives around terminology:

We had research-trained people, we had trained people, and then we layered design thinking on top of that. We have this tripartite thing, that was all driving towards the same end but they were using different languages to basically describe the same thing. And it really took a lot of work, it was [a senior leader] who did a lot of work in navigating a lot of tension between three groups (Provincial Office 2).

I think what might be useful is that when this area of expertise is being discussed, always gently share the definition of IS. Because I might call it something different as an evaluator, and others might call it something else. A program manager might call it something like “program planning”, right? Or others might call it “Plan, Do Study Act cycles”. There are so many different terms that kind of coexist in this space. But once you create that understanding that there might be different terms used, but we're all really interested in this. Then you start to understand there are certain frameworks that help understand those key concepts or constructs that you might want to explore as a group, and then develop that shared mental model of something that we're applying to the situation (Primary Care 1).

### 3.4 Accommodating diversity in a whole-system approach

Alberta's health system had an existing culture of collaboration, where teams valued bringing in partners with different experiences and at different levels of their careers to jointly conduct research. Participants from across the professional groups included in this study appreciated opportunities for transdisciplinary research relationships. Participants overall felt well supported in conducting collaborative research that uses a whole-system approach. Moreover, team members did not feel like their organizations forced them to use a whole-system approach, but rather felt that this approach strengthened their ability to engage with IS:

…[O]ther times, you get really good partnerships, and you get people who say, I've got an idea, and you go, well, let's talk about it. And then together, we co-design, and we co-work and we co-develop an IS for implementation research study. And those are the places where it works really well. And that it gets sponsored by our leadership, and so on, and so forth. It very much depends on the approach of a person who comes in and who wants that support, to do that type of work (Provincial Office 2).

Participants were cognizant that to successfully employ a whole-system approach, they needed to accommodate the different experiences that the different partners brought to IS collaborations. Some participants used a network approach, as one participant described,

We don't require participants to do certain things like they can participate as much or as little you know, any projects, again, are reviewed. It is what I call a whole system network. There are different types of networks, there's bottom up, top down or whole system. Whole system, we found, is usually more sustainable (Research Network 1).

Others described common approaches in quality improvement to support a whole system, including,

I think about our use of things like learning collaboratives, etc. as a way to bring all those diverse stakeholders together … as the start or midway point of implementing some major change, and then having those same stakeholders get together at whatever frequency is needed to live that out (SCN 25).

Despite participants’ commitment to a whole-system approach, fundamental misalignments between the work of implementation science and implementation practice diminished participants’ efforts to collaborate. Specifically, healthcare staff work in contexts characterized by rapid change and urgency. Conversely, researchers are held to highly systematic and rigorous research planning and conduct standards that require more time than health systems can accommodate. Furthermore, research is often considered by health system teams as an activity outside of their mandated quality improvement initiatives. These misalignments complicate meshing academic rigor with health system expectations for rapid change. As one participant recalled,

We worked with a health system impact fellow to put together an ethics application consent form, assess all the risks, you know, a real good research protocol and push back comes back: “You research people are too slow. You're holding us up. This is just QI. We shouldn't be doing any research part of it”. And then a growing realization that actually putting a research framework around that did make the whole thing more robust, because a lot of the stuff that needed to be thought about was thought about up front. But it was a real struggle (Provincial Office 2).

### 3.5 Enabling collaboration

As noted in the Section 2.2 Case Characteristics above, many organizations in Alberta have worked to build infrastructure that enables overall embedded health research, resulting in a strong collaborative research culture. Participants commented on the SCNs and local health research funding programs [e.g., Partnership for Research and Innovation in the Health System Program (50)] as key health research collaboration enablers. Some of the departments or organizations represented by the participants also benefited from their leaders having cross-appointments and/or co-leadership models, facilitating academic–practice partnerships. One intermediary described this benefit as follows:

I think having co-leads from the system as part of our team has definitely increased our ability to sort of have that influence to apply the science and create those science studies in the system (Intermediary 1).

Despite these enablers and the organization-level research infrastructure in place, health system–based teams are still bound to the needs of their organization and must work within available resources:

If we're reaching out to somebody, then this is a project that is a priority for us. Presumably, we've created some time and funding. So the three things for any collaboration are time, funding, and priority. That's because we're part of the healthcare system. We can't just do whatever we want. You know, if I was an independent academic, maybe I have a lot more freedom to sort of explore areas of interest. But as it is, I have areas of interest, but often they are directed by the organization's need (Provincial Office 1).

### 3.6 Facilitating networking

While participants understood the value that IS would bring to implementation practice in Alberta, ongoing silos within and across organizations limited networking opportunities. These silos left teams unable to identify and reach out to potential collaborators across sectors. At times, people could meet through personal and professional connections. As one participant said,

My connections, those were really more through being introduced by colleagues, and not through an organized program. And again, maybe there is something like that, and I just didn't know about it. It was through colleagues in [my faculty], who introduced me to Alberta Health Services folks who are doing work and wanted to collaborate on projects (Academic 7).

People did not suggest that the implementation research community was inaccessible or non-existent, rather it was simply not visible to potential health system teams, as noted by this participant:

I'm not even sure where to go to find the people who know what they're doing in this field, and that are comfortable in working in the messiness of healthcare delivery (Provincial Office 2).

Even participants with cross-appointments at academic centers struggled to find potential partners for implementation research:

There's probably very little broad knowledge of who the IS specialists are. I would say even myself, I am hard pressed to identify people at [my university] that I can refer people to … so I would say that it's kind of word of mouth (SCN 27).

Importantly, participants never mentioned formalized or facilitated networking opportunities existing to help them meet others working in implementation practice or research.

### 3.7 Feedback and mentoring

Academic research partners provided regular input into discussions related to research ideas and methods. They also provided vital access to various research funding streams, all of which were important for career trajectory, as described by one participant:

A lot of [my current IS collaborators] were mentors to me as an early career researcher, so some of these were some of my first opportunities at being co-investigators on a grant, seeing what grant writing is like, being part of quite a big team (Academic 7).

However, formal feedback and mentoring are largely reserved for academic trainees, as health system teams leaned on IS consultants.

Despite the benefits of their potential mentorship, some of the academics interviewed were not actively encouraging their students to consider incorporating IS elements into their graduate programs. When asked if their students were engaged in implementation research partnerships, one academic participant said

No, they were not. Yeah, it really was just me and one of my colleagues doing IS … I can't recall any students who came to me saying that they were interested in IS as a field of study (Academic 7).

Another academic participant commented that students indirectly participated in implementation, through engaged scholarship and intervention research, but none participated as implementation scientists embedded in the health system. This participant also noted that direct partnership opportunities were reserved for later-career academics:

They're developing, they're refining, they're adapting their intervention. They're generating the evidence for the interventions that they would be looking to have adopted in practice with ongoing funding from health systems … but I mean, if technically they're just studying, implementing an intervention, they're not doing that because they're still assistant professors (Academic 5).

### 3.8 Reducing barriers—AbSPORU supports

Using the Partnership Model showed that at the time of writing this article, Alberta's system was well set up to readily embed efficacy and effectiveness research. Infrastructure was also in place to strengthen implementation practice. However, using the Partnership Model to categorize remarks made in the interviews uncovered weaknesses for embedding implementation research, specifically around exchanging knowledge and skills, providing feedback and mentoring, and accommodating diversity. All of these areas affected individual and team abilities to build IS capacity. Without this capacity, teams were not able to participate in embedded implementation research collaborations.

AbSPORU took a whole-system approach to strengthening system IS capacity and capabilities in Alberta. The organization leveraged existing enablers to develop various IS supports to address ongoing challenges at various system levels. AbSPORU's resulting suite of IS-related initiatives are delivered to strengthen IS capacity and infrastructure in Alberta (Table 2). The foundations of this program are presented in the Case characteristics section above. Below are examples of initiatives that also serve to overcome ongoing barriers identified using the Partnership Model in this study (i.e., individual IS capacity, exchanging knowledge and skills, providing feedback and mentoring, and accommodating diversity). Without these capacities and contextual factors in place, people cannot effectively engage in academic–practice partnerships (35).

**Table 2:** AbSPORU's response to reduce existing barriers to embedding IS.

Barriers according to the partnership model constructs	AbSPORU’s response to reduce barriers
Knowledge and skills exchange was most often offered through one-way consultation models instead of embedded, ongoing collaboration. There was also no mechanism to share lessons learned across implementation practice and research teams. IS terminology caused miscommunications, limiting conversations around potential IS partnerships.	AbSPORU provides embedded implementation research support where possible. A Lessons Repository was in development to support sharing implementation lessons learned across siloed teams. A Seminar Series offers a light-touch opportunity for people to learn from experts and to share experiences. The IS Certificate aims to clarify and standardize IS terminology across academic and health system teams.
Accommodating diversity was limited by misalignments between the priorities and work styles of academic and health system partners.	The IS Collaborative provides methodological guidance to strengthen: (1) IS capacity of people working with and on health system innovations (e.g., SCNs); and (2) proposed IS methods to help align rigor and practicality.
Networking opportunities were scarce for implementation support practitioners and implementation scientists.	The transdisciplinary membership of all IS Collaborative groups provides organic networking opportunities.
Feedback and mentoring were reserved for specific types of academic trainees, leaving others, including health system researchers, with little opportunities to build IS capacity.	The IS Certificate was developed to increase IS capacity for academics and health system staff who work to implement health innovations. The IS Collaborative provides IS-specific feedback for health innovation teams looking to incorporate IS into their work.

To build individual and team-based capacity for IS, AbSPORU offers an IS Certificate program, open to academics and health system staff wanting to conduct and use IS in their work. They also offer a monthly IS seminar series that brings international IS experts to discuss different frameworks and approaches they have used to partner and support real-world implementation initiatives. Alberta-based implementation support practitioners involved in the scale, spread, and/or sustainment of health innovations are invited to this series. In addition to capacity-building, the IS Certificate Program and seminar series offer networking opportunities for people working in implementation practice and research.

AbSPORU staff also work to provide mechanisms for knowledge exchange. At the time of this publication, staff were conducting foundational research to inform an online implementation lesson repository. The lesson repository is an effort to directly address the identified inability to share implementation knowledge between teams. The repository will also contain contact information so that potential IS collaborators can reach out to people with similar research interests.

Finally, AbSPORU facilitates a transdisciplinary initiative, called the IS Collaborative, that leverages existing IS expertise to build IS capacity locally (22). The IS Collaborative aims to address some individual and team-level capacity needs and strengthen other organization-level elements to overcome widespread barriers to IS partnerships (e.g., silos, work that is misaligned between research and practice). Specific details of the IS Collaborative are reported by Flynn et al. (22) and on the AbSPORU website (57). The most important element of the IS Collaborative model to note for this article is its transdisciplinary nature. The IS Collaborative includes a Steering Committee and Working Group comprised of Albertans with implementation expertise from sectors across the health continuum. It also includes a Scientific Advisory Board, an international group of leading IS thinkers. Together, these groups offer in-depth IS methodological advice for otherwise research-capable health innovation teams (e.g., SCNs). The transdisciplinary nature of the IS Collaborative supports a whole-system approach to implementation research and practice by providing methodological insight that would help teams develop both rigorous and practical approaches to embedding implementation studies into their health initiatives. The IS Collaborative model is largely consultation-based, but when able, AbSPORU works to find ongoing embedded support that maintains connections with the collaborative. Some participants indicated that consultation models had limited usefulness for conducting implementation research. Nevertheless, AbSPORU proceeded with this model because they did not have sufficient funding to guarantee ongoing embedded support for all IS Collaborative–supported teams. To alleviate this limitation, the IS Collaborative provides consultation to otherwise research-capable teams. Consultants work to build implementation research capacity within these teams, rather than conducting implementation research for them. Consequently, the IS Collaborative works to end reliance on IS consultants over time.

## 4 Discussion

Our findings illustrate that IS partnership considerations go beyond interpersonal factors and include system-wide considerations. Many of the ongoing challenges for IS partnerships uncovered by this study suggest value in further integration between academia and the health system in Alberta. Without this integration, the province misses tremendous opportunities to leverage its provincial learning health system infrastructure to improve implementation methods. Specifically, our results showed that while Alberta has a very strong learning health system infrastructure in place, individuals and teams lack IS capacity, shared language, and communication pathways required to identify potential collaborators and discuss implementation research opportunities. Without these capacities and structures in place, teams cannot negotiate implementation research designs that balance practical needs with scientific rigor. Consequently, these challenges perpetuate misaligned work styles between implementation support practitioners in the health system and implementation scientists that thwart practice–academic partnerships.

### 4.1 Individual and team IS capacity

Our participants emphasized the lack of IS capacity more than any other barrier. Given the extensive enablers in place in Alberta's context, this result verifies other study findings that the lack of IS capacity undercuts the benefits brought from additional research enablers (58). Interview participants had varied levels of capacity and interest even though teams had the same role in the same organization, suggesting that there is no clear mandate for academic or health system teams to engage in IS. An organization-wide mandate to engage in IS is unnecessary, but teams responsible for planning, evaluating, and sustaining change would benefit from IS training as it would help them identify implementation research questions in their work and discuss potential research opportunities with others. In addition, participating in academic–research partnerships strengthens practitioners’ abilities to engage with emerging evidence (35). Engaging in implementation academic–practice partnerships can help implementation practitioners develop important competencies related to ongoing improvement. Specifically, these partnerships can help implementation practitioners to (1) keep abreast of implementation frameworks, strategies, and approaches; (2) become familiar with how these frameworks operate within local contexts; and (3) support implementation improvement cycles. These are core competencies for all implementation practitioners (20) and will be vital for those working in learning health systems’ contexts that aim to strengthen implementation and sustainment methods, such as Alberta Health Services (47).

The closed nature of the IS community has previously been noted as a barrier for engagement in IS partnerships to advance IS (58). Our results suggest that gatekeeping elements may be present in Alberta's IS research community. Supervisors have good reasons to be cautious about when and where to involve trainees in practice–academic partnership; however, the finding that exposure to implementation research partnerships is more easily accessible for later-career researchers is problematic as other studies have found that active mentorship is the key for students to learn how to balance academic and practice priorities and to build networks for future partnerships (17, 21).

The importance of feedback and mentoring goes beyond supporting academic trainees. Access and exposure to IS increases engagement in IS by other partners, including health system teams (58, 59). The IS seminar series and the multi-stakeholder panels in the IS Collaborative deliver this exposure to interested researchers housed in academia or the health system. Other programs that aim to strengthen delivery science in learning health systems have reported positive outcomes from mentorship programs (17, 59). Adding a facilitated IS mentorship program for people throughout the health-research ecosystem could enhance the barrier reduction efforts AbSPORU has already put in motion.

Despite this study uncovering barriers that require additional attention, as described above, AbSPORU's current program already includes important components that are helping to strengthen IS capacity in Alberta. Key AbSPORU contributions include its IS Certificate Program and embedded research services. These supports ensure that health system partners receive more than consultation, which the participants indicated as insufficient. These contributions allow people to acquire and develop key IS skills to use in future partnerships, a key positive impact reported in other embedded research initiatives (60, 61). Consequently, the increased capacity further strengthens the local learning health system (62).

### 4.2 Connecting and working with IS partners

Even though leaders in Alberta's health system have supported investment in infrastructure to support practice–academic partnership, potential implementation research collaborators struggle to find partners with aligned research interests, a common IS barrier (21, 59). In lieu of any networking opportunities, Alberta’s teams turn to personal connections to identify potential partners with IS capacity. Our results corroborate other studies that highlight the importance of cross-appointments for practice–academic partnership (30, 35, 62). Previous studies highlight the flexibility that cross-appointments provide for individual researchers to work in both practical and academic spaces (30, 35). Others point to researchers who also hold leadership positions in health systems and the power this can bring to negotiating research designs that balance rigor and practicality (62). Our study adds the power of non-researcher leaders with cross-appointments. Non-researcher leaders with cross-appointments can be critical for building academic–practice partnership simply because of their knowledge of the systems they work within. These leaders can act as brokers who help overcome the barriers associated with identifying and connecting with appropriate partners.

Beyond personal connections, our results suggest that developing an accessible mechanism to connect with other implementation community members would help address barriers to finding implementation partners. AbSPORU initiated building such a mechanism with a component to allow teams to share lessons learned in previous implementation efforts. As such, this mechanism could address both knowledge exchange and networking gaps that currently exist in Alberta.

Our participants emphasized that finding partners with aligned communication and working styles was as important as finding partners with complementary expertise. Ambiguity in IS terminology created barriers for our participants to engage in discussions around potential implementation research partnerships. The challenges created by loose, unclear, or misused terminology is an important issue to address, as shared language helps build partnerships and ensure that research ideas and evidence are accessible to all the partners (21). Further difficulties emerge in research collaborations because of the misalignment of needs and priorities between researchers and implementation support practitioners. This is a well-documented reality (21, 58, 63, 64) that the IS Collaborative aimed to pre-empt by including scientific and practice-based perspectives in all support provided to innovation teams. Integrating these perspectives was a key activity in early IS Collaborative planning (22). Furthermore, the multiple perspective conversations seemingly became a key strength of the initiative as they helped ensure that the resulting IS advice helps innovation teams to develop rigorous and practical implementation research designs. The IS Collaborative is currently undergoing an impact assessment, which will confirm whether this model of feedback helps increase IS ultimately conducted and used in Alberta's health system.

### 4.3 Enabling IS partnerships

The comments from our health system–based participants align with findings from other studies that fundamental organizational requirements enabling health system teams to engage in implementation research include dedicated time (30), funding (30, 31, 58), and priority or mandate (31, 58, 63, 64). For example, participating health system staff noted that Alberta Health Services encourages them to engage in implementation academic–practice partnerships. However, these health delivery teams were not given protected time or funding for these collaborations. Therefore, teams technically had the permission to engage but the mandate was too weak to successfully inspire implementation academic–practice partnerships. As mentioned above, organization-wide engagement mandates are not necessary, but if organizations want teams to engage in academic–practice partnerships, health system staff require time, funding, and the mandate. Without all three, the low relative priority of these potential partnerships will force teams to decline invitations to engage.

While participants acknowledged the need for funding to engage in implementation research, funding did not emerge as a central barrier to partnership. Indeed, participants cited provincial funding programs (i.e., the Partnership for Research in Innovation in the Health System Program and the Health Innovation Implementation and Spread Fund) (56) as facilitators. Possibly, funding was not considered a key barrier because the interview took place while new IS-specific funding streams were emerging in Canada. Specifically, Canada's federal funding agency, the Canadian Institutes of Health Research, had recently launched its Transforming Health with Integrated Care initiative, which includes an IS team grant component (65). While highly competitive, these new funding streams indicate a growing value of IS and may have helped participants think about barriers beyond funding that affect their partnership abilities.

The rich descriptions by our participants of facilitators and barriers to IS partnerships uncovered strengths and weaknesses throughout the system. As such, our results substantiate other studies that call for a whole-system approach to research capacity-building generally (21, 31) and confirm that developing IS capacity also benefits from a whole-system approach.

The Partnership Model of Whitworth et al. was useful for identifying local parties’ capacity to conduct implementation research and subsequently mobilize the findings. The Partnership Model also helped our research team think through which strengths can be leveraged and weaknesses must be addressed to increase academic–practice implementation partnerships in our local health system. Furthermore, the model helped design implementation support systems. This process of operationalizing the Partnership Model could be useful for other healthcare organizations trying to create conditions to readily embed implementation research and promote IS use in day-to-day implementation processes.

## 5 Strengths and limitations

Our study included participants representing labs, offices, and organizations across Alberta's health-research ecosystem, providing a rich sample of perspectives. Furthermore, the interview data and analysis were both reviewed by all participants, who were invited to share supplementary insights and feedback. This feedback was incorporated into the final analysis and presentation of the results. Nevertheless, trainees were not included in this study, consequently limiting the results to those that would benefit people in later stages of their careers.

Using the Partnership Model strengthened our analysis because of its alignment with our interests of identifying capacity and contextual factors that affect academic–practice partnerships. Specifically, we used the model to explore and understand IS capacity limitations and contextual factors that exacerbate these limitations. The knowledge created by using the Partnership Model helped us think about potentially beneficial capacity-building interventions. However, the model may not have helped capture other contextual factors the limit of use of IS in practice. Furthermore, the Partnership Model would be less appropriate for teams looking to implement specific interventions. In those cases, well-established IS models and frameworks (e.g., Consolidated Framework for Implementation Research) would be more appropriate for exploring implementation barriers and facilitators.

This research assessed a Canadian health system that is situated in a publicly funded, universal healthcare delivery model. As such, the components of the IS Collaborative model may not be transferable to other contexts. Nonetheless, the Partnership Model proved to be a useful tool for assessing the strengths and weaknesses of local embedded research capacity.

## 6 Conclusion

Using the Partnership Model to assess challenges across system levels was a useful exercise, as it helped see what strengths could be leveraged and what interventions could increase Alberta's ability to readily embed IS. The IS Collaborative was built to respond to the challenges identified by providing methodological support and building ways for implementation teams to connect and learn from one another. At the time of this publication, AbSPORU was also delivering other capacity-building programs for individuals as well as developing a cross-sectoral mechanism to share implementation lessons learned. Together, AbSPORU and the IS Collaborative provide insights into developing a whole-system response to the challenges identified in the Alberta context.

## Data Availability

The raw data supporting the conclusions of this article will be made available by the authors, without undue reservation.
